# Microstructure Evolution of TiC Particles In Situ, Synthesized by Laser Cladding

**DOI:** 10.3390/ma10030281

**Published:** 2017-03-11

**Authors:** Yanhui Liu, Jieqiong Ding, Weicheng Qu, Yu Su, Zhishui Yu

**Affiliations:** School of Materials Engineering, Shanghai University of Engineering Science, Shanghai 201620, China; dingjql@163.com (J.D.); quwc2015@outlook.com (W.Q.); xlwsy@hotmail.com (Y.S.); yu_zhishui@163.com (Z.Y.)

**Keywords:** laser cladding, microstructure, TiC, metal matrix composite coating

## Abstract

In this paper, a TiC reinforcement metal matrix composite coating is produced using nickel and graphite mixing powder on the surface ofTi-6Al-4V alloy by laser radiation. The microstructure of the coatings is investigated by XRD, SEM and EDS. Results show that most of the TiC phase is granular, with a size of several micrometers, and a few of the TiC phases are petals or flakes. At the cross-section of the coatings, a few special TiC patterns are found and these TiC patterns do not always occur at the observed cross-section. The even distribution of the TiC phase in the coatings confirms that the convection of the laser-melted pool leads to the homogenization of titanium atoms from the molten substrate, and carbon atoms from the preplace powder layer, by the mass transfer. The characteristics of the TiC pattern confirm that the morphology and distribution of the primary TiC phase could be influenced by convection. Two main reasons for this are that the density of the TiC phase is lower than the liquid melt, and that the primary TiC phase precipitates from the pool with a high convection speed at high temperature.

## 1. Introduction

In situ metal matrix composite (MMC) coatings involve the reinforcement phases being synthesized in a metallic matrix which might be molten by chemical reactions between elements, or between the element and compound, during the coatings process. Compared to ex situ methods, in situ metal matrix composite coatings exhibit the following advantages: greater thermodynamic stability and stronger interfacial bonding, finer size and uniform distribution [1,2,3]. Due to the properties of metal matrix composite coatings, the selectivity of the manufacturing process mainly focuses on various high-energy beam technologies, such as welding arc [4], plasma arc [5], electron beam [6] and laser beam [7]. Among them, laser surface processing has attracted considerable attention to produce MMC coatings as a result of (a) contactless and non-vacuum; (b) high-speed and easy automation; (c) and flexibility for various working conditions with the developing of laser manufacturing technology [7,8,9].

The main characteristics of laser surface processing are high temperature, high speed and continuous production using a dot energy source. Therefore, the manufacturing process has had an important influence on the size and distribution of the reinforcement and the properties of MMC coatings. It has been confirmed in both theory and practice that convection behavior has a strong effect on MMC coatings [10,11,12,13,14]. In particular, the effect of convection on the homogenization of chemical composition [15], surface roughness [16] and geometrical characteristics [10,17,18] has been discussed.

Among different MMCs coatings, TiC reinforcement coatings have shown great potential for metal surface strengthening applications because of appropriate properties such as high hardness and high melting point, as well as excellent thermal and chemical stability. In addition, TiC is particularly suitable for in situ synthesis during non-equilibrium rapid solidification processes (such as laser cladding), owing to the active chemical properties of titanium, and the relatively wide stoichiometric ratio of TiC_x_ [19,20,21,22,23]. Therefore, many research groups have focused on producing in situ TiC reinforcement metal matrix composite coatings. Yu et al. researched TiC/Ti composite coatings by high frequency induction cladding using Ti-graphite mixing powder. Their results showed that the graphite phase remained between the surface of the coating and the substrate, although metallurgical bonds were formed between the coating and the substrate [24]. AIMangour et al. assumed that the fine TiC embryos were formed from the Ti-C-316L liquid system and then rearranged by Marangoni convection, which led to a homogenous distribution of TiC particles in the laser molten pool [25]. Borkar et al. studied the 3D microstructure of TiC in laser-deposited in situ TiC-reinforced nickel matrix composites by three-dimensional reconstruction. They found that the eutectic TiC formed a network linked by primary TiC or graphitic nodules at the nodes [26]. Li et al. investigated the effect of differing Ti/N_b_ ratios in the cladding powder, on the formation mechanism and distribution characteristics of in situ (Ti, N_b_)C particle-reinforced Fe-based composite coatings by laser cladding. Their results indicated that the variation of Ti/N_b_ ratio led to the change of the gap between the formation of Gibbs free energy of TiC and N_b_C, and had significant effects on the growth progress of multiple carbide particles [27].

In this study, A Ni + graphite mixing powder was placed on the clean surface of Ti-6Al-4V alloy and then radiated by a laser beam. The microstructure of the single track coatings, particularly the microstructure of TiC, was analyzed. Because the only source of titanium atoms in TiC was the melted substrate due to convection in the laser melted pool, the effect of the convection behavior on the solidification of a laser melted pool was studied.

## 2. Experiments

**Figure 1:**
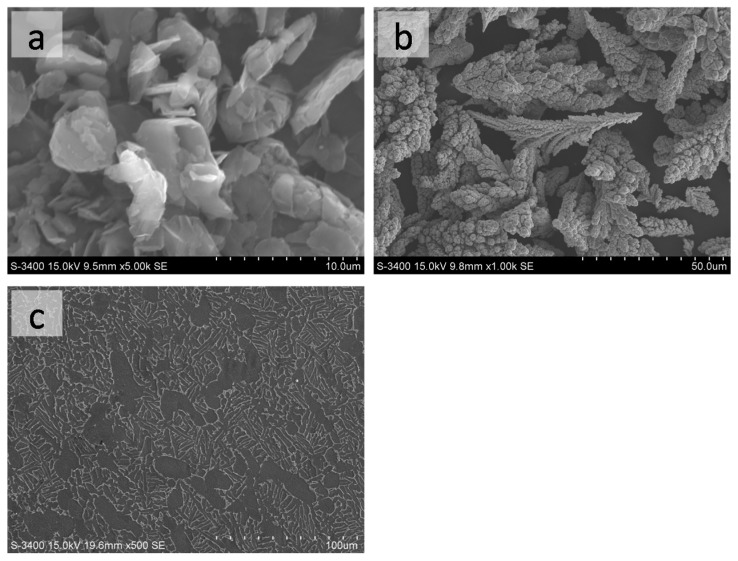
The SEM images of the laser cladding powder and the substrate. (**a**) Graphite powder; (**b**) Nickel powder; (**c**) Substrate.

Commercial-grade graphite (3500 meshes, 99.0% purity, Figure 1a) and nickel powder (200 meshes, 99.5% purity, Figure 1b) was used as the additional powder for the laser treatment. The powders were mixed with 10.0% carbon by mechanical grinding in grinding cans with gradation agate balls. The substrates were Ti-6Al-4V alloy, with a size of Φ 50 mm × 10 mm. The substrate was observed to be typical of α + β titanium alloy (Figure 1c).

The experimental design was based on a previous study [28]. The mixing powder was preplaced evenly on the clean surface of the substrate at a thickness of approximately 1.2 mm. The powder layer was pressed at room temperature to eliminate residual air and to avoid the layer being blown away by the shielding gas flow. Single-channel coatings were produced by a 5 kW continuous-wave CO_2_ laser system under an argon shielding gas using the following processing parameters: laser spot size 4 mm, laser scan speed 5 mm/s, and laser power 2.4 kW. The energy balance equation of the laser beam was a Gauss beam distribution.

After laser cladding, samples were prepared by electrospark wire-electrode cutting, and standard mechanical grinding and polishing. It should be noted that samples were not chemically etched. Microstructural morphology and chemical composition were analyzed by a Hitachi S-3400 scanning electron microscope (SEM) with a genesis-type X-ray energy dispersive spectrometer (EDS) attachment. Phase identification was carried out with a Philips X’Pert PRO X-ray diffractometer (XRD) using Cu Kα radiation (λ = 1.54 Å).

## 3. Results and Discussion

**Figure 2:**
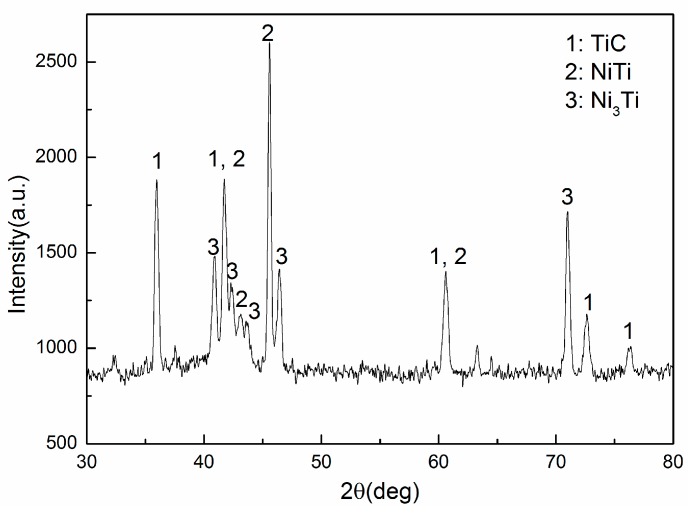
The XRD spectrum of the laser cladding coatings.

From XRD patterns of a single-track coating formed by laser cladding, strong diffraction peaks corresponding to TiC, NiTi, and Ni_3_Ti could be clearly identified (Figure 2). Because the powder layer was a mixture of nickel and graphite and the substrate was Ti-6Al-4V alloy, the XRD results indicated that the Ti atoms of the substrate had been mixed with C atoms and Ni atoms. TiC and Ni-Ti intermetallic were subsequently synthesized in the laser melted pool. Studies have confirmed that the Marangoni convection of the laser melted pool plays a key role in mass transfer and chemical composition homogenization in the laser melted pool [10,11,12,13,14]. Therefore, the XRD results indicated that the Marangoni convection occurred in the laser melted pool and led to the homogenization of the chemical composition of both the preplaced layer and the melted substrate.

**Figure 3:**
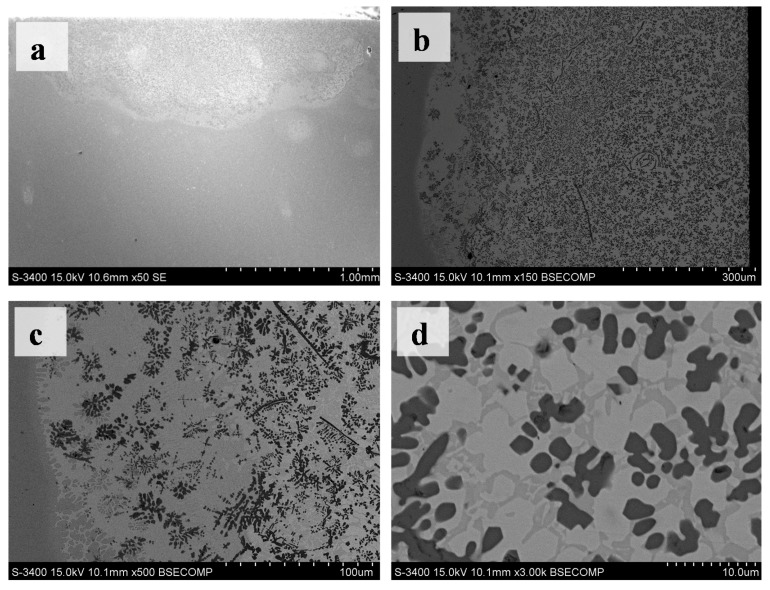
SEM images of the cross sections of the single-channel coatings. (**a**) The macro-morphology of the coatings; (**b**) The microstructure of the coatings; (**c**) The bottom microstructure; (**d**) The middle microstructure.

The bonding line between the single-channel coatings and the substrate was an undulating curved line, which was believed to be caused by the heat transfer effect of melted pool convection (Figure 3). It was clear that a significant amount of the black particle phase was uniformly distributed in the single-channel coatings (Figure 3b). Because the images of Figure 3 were backscattered electron scanning images, the black reinforcement phase in Figure 3b should be TiC. The TiC phase was reduced at the bottom of the coatings, with a thickness of about 100 μm (Figure 3c). In addition, it was clear that a grey white phase began to appear about 100 μm from the bonding line. According to the XRD results and the principle of backscattered electron scanning, the grey white phase was assumed to be the rich nickel phase (Ni_3_Ti). Figure 3d was the magnified image of the middle of the coatings. Three grey levels represented three phases associated with three crystal structure in Figure 2 (Figure 3). Table 1 was the EDS point analysis data from three grey levels in Figure 3d. The EDS results agreed with the microstructure of the coatings (Table 1). It should be noted that the carbon content in the grey white zone and in the grey zone for Table 1 was not calculated.

**Figure 4:**
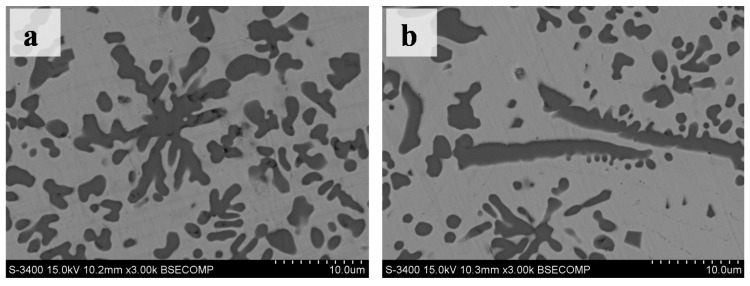
SEM images of the TiC phase at the cross sections of the coatings. (**a**) The TiC petals; (**b**) The TiC flake.

**Figure 5:**
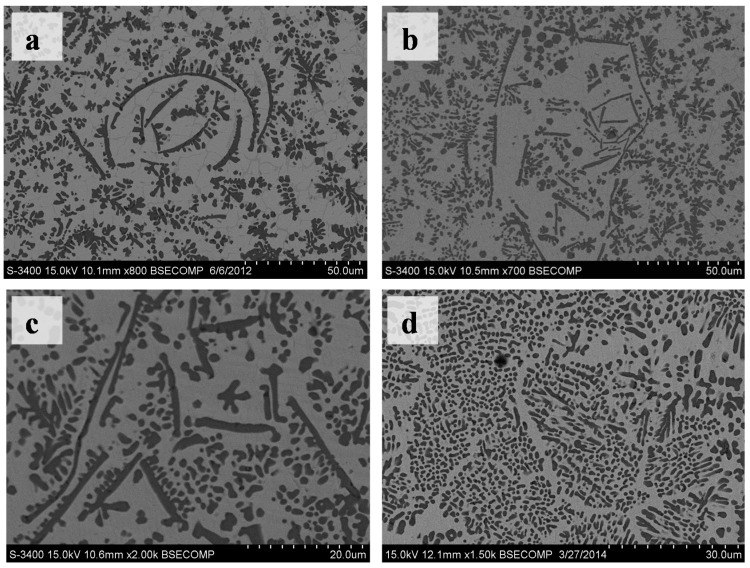
SEM images of the TiC patterns at the cross sections of the coatings. (**a**) The spiral-like TiC pattern; (**b**) The polygon-like TiC patterns; (**c**) The worm-like TiC patterns; (**d**) The leopard-like TiC patterns.

In this experiment, most of the TiC phase was granular with a size of about several micrometers (Figure 3d). In addition, a few TiC phases were petals or flakes at the cross-section of the coatings (Figure 4a,b). Furthermore, different TiC patterns made up of granular TiC particles, flakes and petals were present in the cross-section of the coatings (Figure 5). For example, there was a group of arc-shaped TiC particles with a swirling distribution composed of the spiral-shape TiC pattern (Figure 5a). The fine granular TiC particles were distributed only at the convex side of the arc-shaped TiC particles (referred to as the worm-like TiC patterns in Figure 5c). The polygon-like TiC patterns in Figure 5b were similar to the spiral-shape TiC pattern in Figure 5a. The fine granular TiC particles were distributed only on one side of the line-shaped TiC particles, while the worm-like TiC patterns did not form any special patterns (Figure 5c). The distribution of the microstructures (referred to as leopard-like TiC patterns) was similar to eutectic ledeburite in the white cast iron (Figure 5d). Some strip zones without the TiC phase looked similar to the crystal grain boundary in the microstructures. It should be noted that these special TiC patterns did not always occur in the observed cross-sections, although one or more TiC patterns could often be found at the observed cross-section in this research.

From to the XRD results (Figure 2) and microstructure analysis (Figure 3, Figure 4 and Figure 5), this research proposed the following assumptions: (a) the graphite was completely used for the TiC synthesis; (b) α-Ti phase, β-Ti phase and γ-Ni phase did not appear in the coatings; (c) the coatings were composed mainly of the TiC, NiTi, and Ni_3_Ti phases. Though the composition ratio of the Ni-Ti-C system in these coatings could not be known precisely because the titanium content from the substrate by laser cladding could not be accurately measured, the nickel content was higher than the titanium content because the Ti atoms would have been utilised for TiC synthesis and subsequently for Ni-Ti intermetallic synthesis, under the same conditions.

Bandyopadhyay et al. determined the isothermal sections to be at 900, 1070, 1260 and 1700 °C [28]. According to the above analysis and the latest binary data about the Ni-Ti-C system [29], the microstructure of the coatings in this research should have been the TiC_1-x_ + Ni_3_Ti + NiTi. We agree that the microstructure in Figure 5d was the eutectic microstructure. Thus, the probable eutectic reaction equation according to the reaction scheme in [29] was the following:

L ⇋ TiC_1-x_ + Ni_3_Ti (1367 °C) (1)

L ⇋ TiC_1-x_ + NiTi (1280 °C) (2)

L ⇋ TiC_1-x_ + NiTi + Ni_3_Ti (1124 °C) (3)

L ⇋ NiTi + Ni_3_Ti (1120 °C) (4)

**Figure 6:**
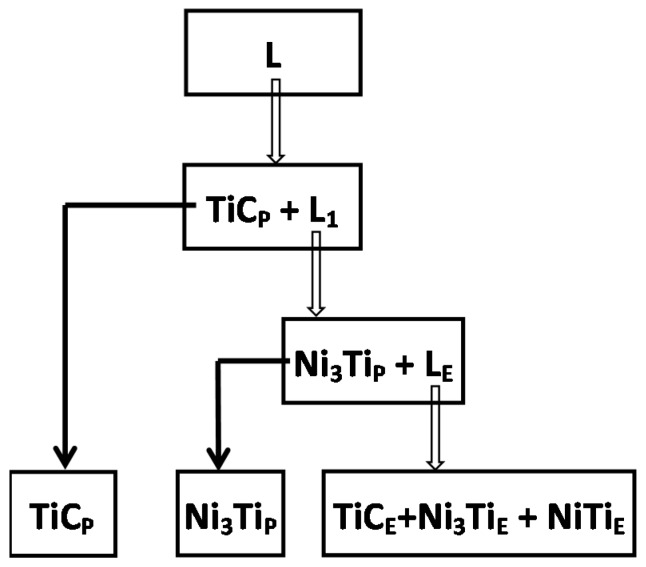
Solidification processes of the Ni-Ti-C system in a laser-melted pool. TiC_P_: Primary TiC; TiC_E_: Eutectic TiC; Ni_3_Ti_E_: Eutectic Ni_3_Ti; TiNi_E_: Eutectic NiTi.

Because the area of the Ni_3_Ti phase was bigger than the NiTi phase (Figure 3d and Figure 5a), the most probable eutectic reaction equation is Equation (1) at the eutectic reaction temperature for this study. The TiC morphology was affected by the convection behaviors of the laser melted pool (Figure 5a,b). This indicated that the TiC phase had grown with strong convection in the high temperature laser melted pool. Therefore, the probable phase that exists above 1700 °C should be TiC_1-x_ + L [26,29]. In conclusion, the probable solidification processes of the Ni-Ti-C system in this research are shown (Figure 6).

**Figure 7:**
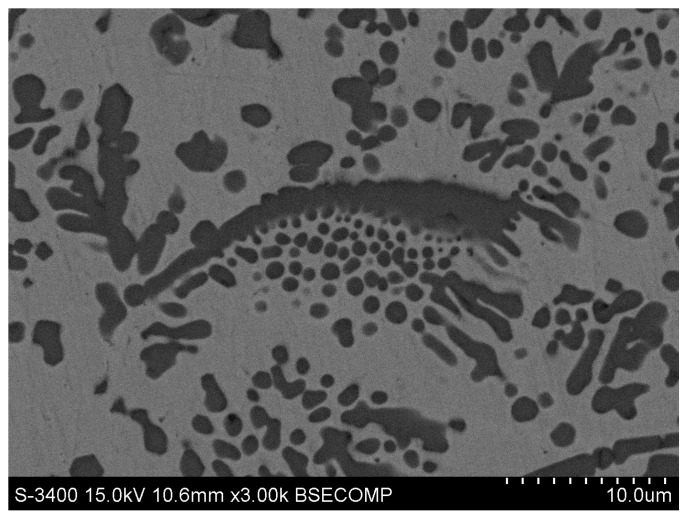
The TiC microstructure from primary to eutectic.

Many studies have indicated that the convection of the laser melted pool is complex and volatile. Therefore, the distribution and morphology of the TiC particles is also variable due to convection effects. This is because low density primary TiC particles can rise above 1700 °C in the high density liquid pool with high speed convection [28,29]. Therefore, it was reasonable for the morphology and pattern of TiC particles to have occurred (Figure 5a). In addition, the morphology and pattern of TiC particles in Figure 5b,c should be the variation of those in Figure 5a from the decreasing convection speed and the decreasing pool temperature. Also, a eutectic microstructure formed when the chemical composition was homogenized while the melt was not under convection (Figure 5d). Wang et al. found that the TiC rose up to the surface of graphite in the Cu-Ti melts under quasi-static conditions [30]. The TiC particles would subsequently cease rising, separate from the graphite and melt. Thus, TiC with a wide range of sizes and morphologies was obtained. The results of Wang et al. supported our discussion with regards to Figure 5d. A typical transition microstructure from primary to eutectic was shown in Figure 7.

Other significant characteristics of the primary TiC were that fine TiC particles were distributed only at one side of the bigger TiC particles (Figure 5a–c and Figure 7). As an example, the chemical composition changes of the TiC pattern in Figure 7 were investigated by EDS point analysis to explore why the fine TiC particles were distributed only at one side of the bigger TiC particles.

**Figure 8:**
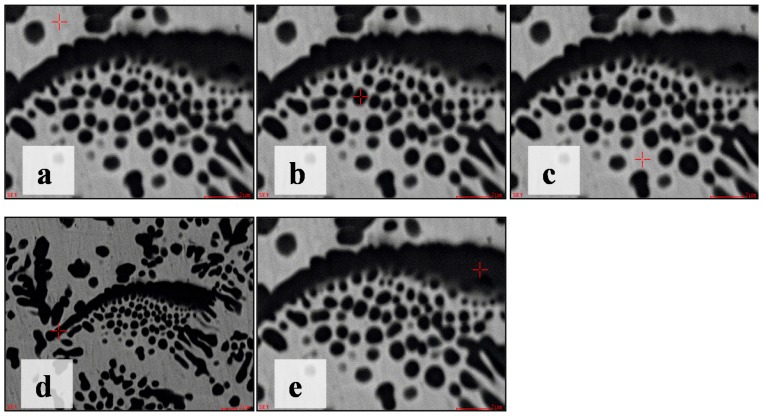
The diagram of EDS point analysis for Figure 7. (**a**) EDS point A; (**b**) EDS point B; (**c**) EDS point C; (**d**) EDS point D; (**e**) EDS point E.

Though the EDS analysis of carbon was not accurate, the qualitative level of carbon content was determined [23]. The carbon content was lowest at point A in Figure 8a, and especially lower than point C (Table 2). This should be an important factor for eutectic TiC formation under the flake TiC particles (Figure 7). Though all three points contained TiC particles, the nickel content was 1.58% at point E but 7% at point B, and 7.82% at point D. The most probable reason for this was that there was some error between the EDS point analysis size and the TiC particle size. The TiC particle size at point B and point D was smaller than the other EDS point analysis sizes (Figure 8). This same issue also led to higher titanium content at point C. We assumed that flake TiC particle rose first in the convection of the laser melted pool, and that its shape and growth impeded the chemical composition homogenization of the rest of the liquid melt. The eutectic TiC would precipitate in the higher carbon zone. If sufficient convection in the zone of Figure 7 had occurred, the flake TiC and the eutectic TiC may have developed similar TiC patterns to those of Figure 5a. The distribution and shape of fine TiC particles in Figure 7 agreed with TiC growth mechanisms from in situ synthesis [30].

## 4. Conclusions

A TiC-reinforced Ni-based composite coating was produced on the surface of Ti-6Al-4V alloy when the nickel and graphite mixing powder layer was radiated by a laser beam. At the cross-section of the coatings, most of the TiC phase was granular, with a size of several micrometers, and a few TiC phases were in petal or flake composition. In addition, some TiC particles formed a number of special TiC patterns; this did not always occur in the observed cross-sections. The even distribution of the TiC phase confirmed that the mass transfer of the convection in the laser melted pool led to the homogenization of titanium atoms and carbon atoms. However, the morphology of the TiC phase and the characteristic of TiC patterns indicated that the morphology and distribution of the TiC phase were also influenced by the convection of the laser melted pool, especially that of the primary TiC phase. This was due to the lower density of the TiC phase compared to the liquid melt, and the primary TiC phase precipitating from the pool with high convection speed at high temperature.

## Figures and Tables

**Table 1:** EDS point analysis data from three grey levels in Figure 3d.

Test Zone	Ni (at %)	Ti (at %)	C (at %)	Al (at %)	Matrix
Grey white zone	75.95	20.06	-	3.99	ZAF
Grey zone	54.39	42.16	-	3.45	ZAF
Black zone	2.63	40.31	57.06	-	ZAF

**Table 2:** EDS point analysis data in Figure 8.

No.	C (at %)	Ti (at %)	Ni (at %)	Al (at %)	Matrix
a	37.09	9.37	49.57	3.97	ZAF
b	59.96	32.63	7.00	0.42	ZAF
c	54.25	24.73	19.81	1.21	ZAF
d	64.58	27.05	7.82	0.56	ZAF
e	63.19	35.23	1.58	-	ZAF
